# Supplementary material for: Tumor- and Fibroblast-Derived Cell-Free DNAs Differently Affect the Progression of B16 Melanoma In Vitro and In Vivo
Source: Int J Mol Sci. 2024 May 13;25(10):5304. doi: 10.3390/ijms25105304 (PMC11120878; doi:10.3390/ijms25105304)
Supplement: Supplementary file 1 [file ijms-25-05304-s001.zip › ijms-2962019-supplementary.pdf]

Materials and Methods

Measurement of 8-Oxo-2′-deoxyguanosine in the cfDNA

The levels of 8-Oxo-2′-deoxyguanosine in the cfDNA extracted from condition media of B16 and L929 cells using ELISA General 8-Oxo-2′-deoxyguanosine Kit (ABclonal, Massachusetts, USA) following the manufacture's protocols.

Results

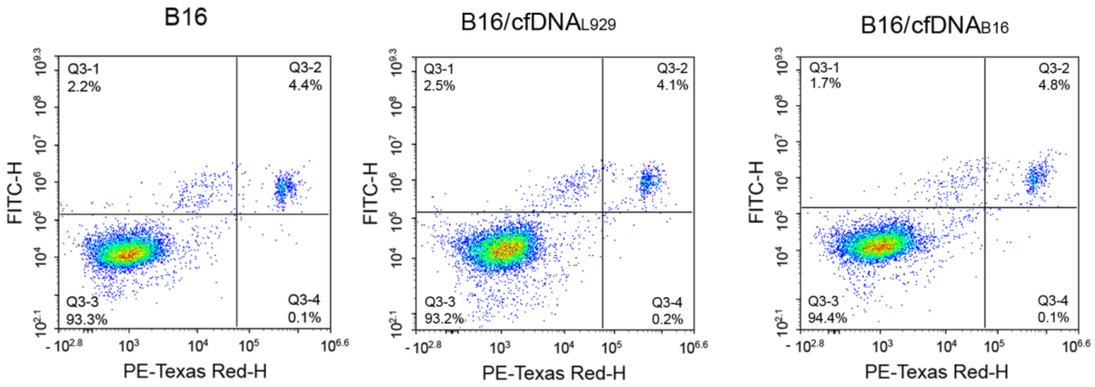

Figure S1. The effect of cfDNA on the apoptosis of B16 cells**.** B16 cells were incubated in presence of cfDNA$_{B16}$ or cfDNA$_{L929}$ at concentrations 100 ng/mL for 24 h. The results of flow cytometry.

Table S1. The effect of cfDNA$_{B16}$ and cfDNA$_{L929}$ treated with RNase A, on viability and migration of B16 cells.

	**B16**	**B16 incubated with cfDNA**			
		**cfDNA$_{B16}$**		**cfDNA$_{L929}$**	
		**-**	**+ RNase A**	**-**	**+ RNase A**
**Concentration of cfDNA, ×10³ ng/mL**	-	150 ± 35	142 ± 42	56 ±12	51 ± 17
**Viability, %**	100 ± 11	81 ± 11	83 ± 12	121 ± 14	118 ± 18
**Migration, %**	100 ± 12	62 ± 19	64 ± 18	119 ± 19	116 ± 15

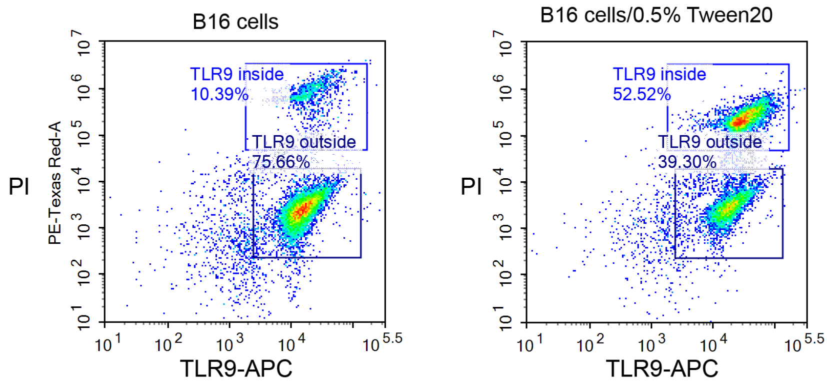

Figure S2. Visualization of TLR9 receptors on B16 cells when combined with PI staining. Flowcytometry data. On the left are intact B16 cells, on the right are cells treated with 0.5% Tween20. An increase in the accumulation of PI-stained cells was shown, without a change in anti-TLR9 staining. Anti-TLP9 staining was also observed in cells not stained with PI (population «TLR9 outside»).

Table S2. Primer sequences used for qPCR.

Name	Forward	Reverse
B1_mus1	AGGCGGATTTCTGAGTTCAA	GAGACAGGGTTTCTCTGTGTAG
B1_mus2	GAGACAGGCGGATTTCTGAGT	TGTAGCCCTGGCTGTCCT
L1_mus1	GCCAGGTATCTGTGCATCTT	ACTCTAGCTCTCTCCTGAGTTT
L1td1_orf1	GAACCAAGACCACTCACCATCA	CCCTGGACTGGGCGAAGT
L1td1_orf2	GCGGTTCCTCAGAAAATTGG	TGCCCAGGAGAGGTATTGCT
Myc_ex1	GAAGGCAGCTCTGGAGTGAG	CTGATGTTGGGTCAGTGCA
Myc_ex2	TCCTCAAACGATGCCTTCCC	GGTGCATCCTCTCACTCTCCA
Myc_ex3	CGACTACGACTCCGTACAGC	CCAGATATCCTCACTGGGCG
Ras	GCTCTGTGTCCATCTACTCATC	AGCCATCAAAGTCTCCTTATCC
mt-Co3	CAGGATTCTTCTGAGCGTTCTAT	ACTTAACCCTCTAGAAGTCCCA
mt-Nd3	AATGCGGATTCGACCCTAC	CTTCTACTTCCACTACCATGAGC
Gapdh	AATGGTGAAGGTCGGTGTG	ACAAGCTTCCCATTCTCGG

Table S3. Primer sequences used for RT-qPCR.

Name	Forward	Reverse	Probe
Actb	TATTGGCAACGAGCGGTTCC	TGGCATAGAGGTCTTTACGG	CCAGCCTTCCTTCTTGGGTATGGAATCC
Braf	AGCGGGAAAGGAAGTCATC	AGTTCCAAATGACCCAGATCC	TCTGTCCATCAGGAATCTCCCAGTCA
Fos	CCTTTGTCTTCACCTACCCTG	CTTGCCTTCTCTGACTGCTC	TGACTCCTTCCCAAGCTGTGCC
Jun	TTGTTACAGAAGCAGGGACG	GTCGTAGAAGGTCGTTTCCATC	AGGCTAACCCCGCGTGAAGT
Kras	GGAGTACAGTGCAATGAGGG	CCATAGGCACATCTTCAGAGTC	AGAAAGCCCTCCCCAGTTCTCATGTA
Myc	GCTGTTTGAAGGCTGGATTTC	GATGAAATAGGGCTGTACGGAG	CGTAGTCGAGGTCATAGTTCCTGTTGGT
Raf1	GGGTTTTCTTGCCGAATAAGC	TCTTACCTTTGTGTTCCTGGAG	ACTGTGGTCAATGTGCGGAATGGA
Trp53	TGGAAGACAGGCAGACTTTTC	GATGGTAAGGATAGGTCGGC	AGCGTGGTGGTACCTTATGAGCC
Raf1	GGGAAATAGAAGCCAGTGAGG	TGAAGTTGCTCTGGAGTTGG	GGGAAATAGAAGCCAGTGAGG
Atp5f1b	CCGTGAGGGCAATGATTTATAC	GTCAAACCAGTCAGAGCTACC	AACGCTACCTTGGAAGTGGCATCT
Atp5If1	ACGAGAAAAGGCTGAAGAGG	ATGGCGTTCAATTTGCTTCTG	TCATGGTGTTTCCTCAGGGCAGC

Ccnd1	GCCCTCCGTATCTTACTTCAAG	GCGGTCCAGGTAGTTCATG	TCCTCACAGACCTCCAGCATCCA
Cdc42	CATGTCTCCTGATATCCTACACAAC	TGTCATAATCCTCTTGCCCTG	ATGGCTCTCCACCAATCATAACTGTGAC
Cdk4	CAGTCTACATACGCAACACCC	GTCGTCTTCTGGAGGCAATC	TTTTCCCCAACTGGTCGGCTTCA
Cdk6	TGACGAACTAGGCAAAGACC	GTTGGATGGCAGGTGAGAG	TTGGAAGTACGGGTGATTCAGGGC
Cdkn2a	GTGCGATATTTGCGTTCCG	TCTGCTCTTGGGATTGGC	TGGGTGCTCTTTGTGTTCCGCT
Coq10a	CTCATAGTCGTTCCTTCATGGG	CCTCAAACATCTCCTGCATTG	CTACGCTCCGAGTAAGCCTTTCGC
Cox6c	GAAGGCTCTCCGTTTCTCC	AGCAATATGAACCCGCAGAC	AGGACGTTGGTGTAGAGGACATTGG
Cycs	AAGGGAGGCAAGCATAAGAC	ATTCTCCAAATACTCCATCAGGG	ACCAAATCTCCACGGTCTGTTCGG
Hmgb1	ACAAGGCCCGTTATGAAAGAG	TTTTGGGCGATACTCAGAGC	TCAAGGATCCCAATGCACCCAAGA
Icam	GCAGAGGACCTTAACAGTCTAC	TACTTGGCTCCCTTCCGAGACCT	TACTTGGCTCCCTTCCGAGACCT
Rac1	TGCTTTTCCCTTGTGAGTCC	TCAGCTTCTCAATGGTGTCC	TCCGTGCAAAGTGGTATCCTGAAGTG
Rhoa	TGAGCCTTGCATCTGAGAAG	TTACCACAAGCTCCATCACC	TGTACAACTGCATCCCAGAACCTGTG
Stat3	GGCACCTTGGATTGAGAGTC	CGAAGGTTGTGCTGATAGAGG	AACGTGGCATGTGACTCTTTGCTG
Dffb	CCAAGCTAACCAAGGAGTCAG	GGAAGCTGTAACTCCTGGAAC	ACATCGGGCTAGGATCTGAACTAAGGT
Dnase1	GATGTCCAATGCTACCCTCTC	CGGTAGGTGTCAGGTTTGTC	CGATTGAGTTCATCCAGGAGCTTCCC
Dnase1l1	CGCAGCACATACAAGGAAAAG	GGAAGAGTTTTGCTAGGGAGAG	CCGAGAACCATTTGTGGCCCATTT
Dnase1l2	ATGGTTTGGTGTTACTCTGGG	CTCTGAACATTGAAGGCTCCA	CGAGGTTTTCTGCTTTTGTCATGGGTTG
Dnase1l3	ACTTCGTGATTGTCCCCTTG	GCGTTGAAATCACCCATGAAG	AACTCCCGAGACCTCCGTTAAAGAGA
Dnase2a	CAAGACGGTGTAGGGTACATC	GAGGTTGGTCGTTGTAGAGTAG	TGGCTGGAGTTCTTTCGGTACAATGG
Dnase2b	TGCTGAGACATCCAACCTTAC	GTGTCAGGTCTTTCTCAGGC	CGGGTCGGTTCACTTTTGCTGTT
